# Local News

**Published:** 1885-06

**Authors:** 


### LOCAL NEWS.

The Fifteenth Annual Commencement Exercises of the Woman's Medical College were held in the Grand Opera House, April 21st.

Degrees were conferred on twenty-two young women by the President, Professor W. H. Byford, M. D., accompanied by an appropriate address.

The Secretary, Professor Marie J. Mergler, distributed the prizes as follows:

The Rosa Engert Prize, for best examination in Medical Microscopy, to Anna Dennis Gloss and Nettie Derexa Morey.

The prize offered by The Woman's Christian Union for the best paper on the Physiological Action of Alcohol, and its Therapeutic Use, to Anne C. Burnet.

The address to the graduating class was delivered by Miss Ada C. Sweet, of Chicago. The subject was "The Age of Humanity."

The Alumnae Association of the College held its annual meeting after the exercises and elected officers and transacted the usual business.

In the evening, a reception was given to the faculty, former teachers, alumnae and graduating class, by Professor and Mrs. Charles Warrington Earle, at their residence.

---

The members of the executive committee of Presbyterian Hospital have decided to enlarge this new hospital, by building a five-story brick building adjoining the north side of it. It is expected that work on the new building will be begun at once.

